# The Key Gene Expression Patterns and Prognostic Factors in Malignant Transformation from Enchondroma to Chondrosarcoma

**DOI:** 10.3389/fonc.2021.693034

**Published:** 2021-09-10

**Authors:** Junqing Wu, Yue Huang, Chengxuan Yu, Xia Li, Limengmeng Wang, Jundong Hong, Daochao Lin, Xiaoping Han, Guoji Guo, Tianye Hu, He Huang

**Affiliations:** ^1^Bone Marrow Transplantation Center, The First Affiliated Hospital, Zhejiang University School of Medicine, Hangzhou, China; ^2^Institute of Hematology, Zhejiang University, Hangzhou, China; ^3^Zhejiang Province Engineering Laboratory for Stem Cell and Immunity Therapy, Hangzhou, China; ^4^Liangzhu Laboratory, Zhejiang University Medical Center, Hangzhou, China; ^5^Department of Colorectal Surgery and Oncology, Key Laboratory of Cancer Prevention and Intervention, Ministry of Education, The Second Affiliated Hospital, Zhejiang University School of Medicine, Hangzhou, China; ^6^Zhejiang University City College, Hangzhou, China; ^7^Department of Orthopaedic Surgery, Shulan (Hangzhou) Hospital, Zhejiang Shuren University Shulan International Medical College, Hangzhou, China; ^8^School of Basic Medical Sciences, Zhejiang University School of Medicine, Hangzhou, China; ^9^Center for Stem Cell and Regenerative Medicine, Zhejiang University School of Medicine, Hangzhou, China; ^10^Zhejiang Provincial Key Lab for Tissue Engineering and Regenerative Medicine, Dr. Li Dak Sum & Yip Yio Chin Center for Stem Cell and Regenerative Medicine, Hangzhou, China

**Keywords:** enchondroma, chondrosarcoma, epithelial to mesenchymal transformation, VEGFA-VEGF2R signaling pathway, T cell immunity, angiogenesis, prognostic factor

## Abstract

Enchondroma (EC) is a common benign bone tumor. It has the risk of malignant transformation to Chondrosarcoma (CS). However, the underlying mechanism is unclear. The gene expression profile of EC and CS was obtained from Gene Expression Omnibus (GEO) database. The differentially expressed genes (DEGs) were identified using GEO2R. We conducted the enrichment analysis and constructed the gene interaction network using the DEGs. We found that the epithelial-mesenchymal transition (EMT) and the VEGFA-VEGF2R signaling pathway were more active in CS. The CD8^+^ T cell immunity was enhanced in CS I. We believed that four genes (MFAP2, GOLM1, STMN1, and HN1) were poor predictors of prognosis, while two genes (CAB39L and GAB2) indicated a good prognosis. We have revealed the mechanism in the tumor progression and identified the key genes that predicted the prognosis. This study provided new ideas for the diagnosis and treatment of EC and CS.

## Introduction

EC is one of the most common benign bone tumors occurring in the medullary space of bone (1). Ollier disease and Maffucci syndrome are subtypes of EC (2). CS is the second most common malignant bone tumor after osteosarcoma, accounting for 20% of the bone sarcomas (3). CS is divided into three grades based on histopathology. The risk of malignant transformation from EC to CS, especially in the Ollier disease and Maffucci syndrome, is as high as 50% (4). Therefore, it is important to understand the underlying mechanism of the malignant transformation.

Though disrupted signaling pathways play roles in both EC and CS, CS has its own unique characteristics (4). However, the key biomarkers involved in the metastasis are still unknown.

High-throughput gene detection technologies are promising tools with a wide range of clinical applications (5). Integrating and analyzing the data in the public database provides an opportunity to reveal the key regulators.

In this study, we used the data GSE30835 from GEO. We identified the DEGs among normal, EC, and CS samples. We performed the gene enrichment analysis to explore the key regulatory pathways. Finally, we proposed some markers that predicted the metastasis. These results may help diagnosis and treatment.

## Materials and Methods

### Microarray Data

The gene expression profile GSE80835 was obtained from the GEO database. The microarray data was established on the GPL6884 platform (Illumina HumanWG-6 v3.0 expression bead chip). There were 6 normal controls (growth plate and cartilage), 7 EC samples, and 14 CS samples. In addition, two other gene expression profiles, GSE17679 and GSE21122, were obtained. GSE17679 contained 64 Ewing sarcoma samples and 18 normal controls. GSE21122 contained 26 leiomyosarcoma samples and 9 normal controls.

### DEGs Identification

GEO2R was used to identify DEGs. When comparing normal control and patient samples, genes with p < 0.01 and ∣logFC∣>1 were considered DEGs. In the comparison between EC and CS samples, genes with p < 0.05 were considered DEGs Graphpad Prism was used to display the volcano plot, and R package “pheatmap” was used to display the heatmap.

### Metascape Enrichment Analysis

Enrichment analysis was performed by using the online tool called Metascape (http://metascape.org). The enrichment terms included GO biological processes, Biocarta gene sets, Hallmark gene sets, Reactome gene sets, KEGG pathway, Wikipathways, and Canonical pathways.

### Protein-Protein Interaction Network

The Protein-Protein Interaction (PPI) network was constructed using the online tool called STRING (http://string-db.org). We selected the top 50 DEGs in patient samples. Among the 50 upregulated genes, 46 were protein-coding genes, and 36 interacted with each other.

### Gene Set Enrichment Analysis

The Gene Set Enrichment Analysis (GSEA) tool was downloaded from the Broad Institute (http://software.broadinstitute.org/gsea). The analysis showed the differences between EC and CS, with statistical significance determined by 1,000 gene set permutations. The enrichment maps with the enrichment scores were generated for visualization of the results.

### Weighted Correlation Network Analysis

The Weighted Gene Co-expression Network Analysis (WGCNA) was used to verify the gene modules in EC and CS. The most relevant modules were selected for further analysis. Cytoscape was applied to establish the network using the top-weighted genes. Metascape enrichment analysis was performed using the genes involved in the modules with the threshold of 0.02 in WGCNA.

### Survival Analysis

We chose the sarcoma datasets in GEPIA (http://gepia.cancer-pku.cn/index.html), which used TCGA data to explore the prognostic potential of certain genes. The project ID of sarcoma datasets in GEPIA including TCGA-SARC, GENIE-MSK, GENIE-DFCI and CMI-ASC. PrognoScan (http://dna00.bio.kyutech.ac.jp/PrognoScan) was used to verify the relation between the gene expression levels and the distant recurrence-free survival using sarcoma dataset GSE30929. The p <0.05 was considered statistically significant.

## Results

### DEGs Reveal the Characteristics of EC and CS

**Figure 1:**
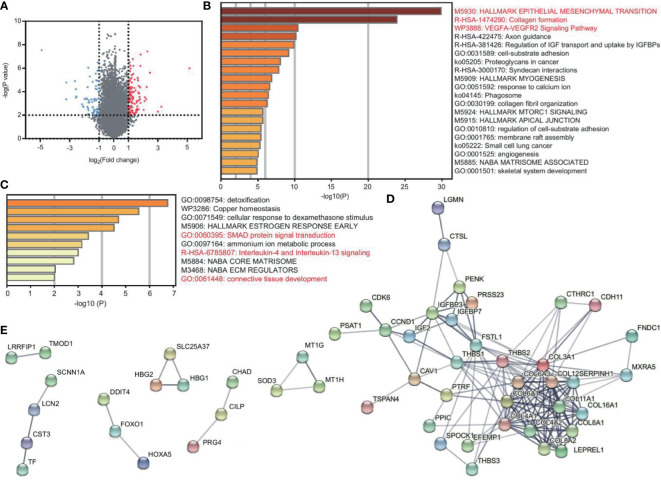
DEGs reveal the characteristics of EC and CS. **(A)** Volcano plot of DEGs between patients (EC and CS) and normal controls. Red and blue dots represent the highly and lowly expressed genes in patients, respectively. **(B, C)** Metascape enrichment analysis for viewing the enrichment terms of upregulated **(B)** and downregulated **(C)** genes in patients. The color shows the p value. **(D, E)** PPI network of the upregulated **(D)** and downregulated **(E)** protein-coding genes in patients.

To explore the DEGs between patients and healthy controls, GEO2R was performed with the criterion of p < 0.01 and ∣logFC∣>1. We found 89 upregulated genes and 52 downregulated genes (**Figure 1A** and **Supplementary Table 1**). We applied Metascape enrichment analysis to identify the altered biological functions. **Figure 1B** and **Supplementary Table 2** show the enriched terms of the upregulated genes. We found that these genes were most related to EMT, collagen formation, and the VEGFA-VEGFR2 signaling pathway. EMT plays a central role in tumor progression, and the VEGFA-VEGFR2 pathway indicates tumor angiogenesis (6, 7). Collagen formation is the basic feature of solid tumors (8). The activation of EMT and angiogenesis were also found in other sarcomas (**Supplementary Figure 1** and **Supplementary Table 3**).

We used STRING to construct the PPI network to confirm the relationship between genes. Among the top 50 upregulated genes, there are 46 protein-coding genes, of which 36 are closely related (**Figure 1D**). In the network, collagen genes, such as COL16A1, COL6A3, and COL8A2 have interacted. They contribute to both EMT and collagen formation. In addition, FNDC1 and FSTL1 are also EMT-related genes. CAV1 is one of the core genes in the VEGFA-VEGFR2 pathway and collagen formation (**Supplementary Table 2**).

The downregulated genes are enriched in the terms of SMAD protein signal transduction, IL-4 and IL-13 signaling, and connective tissue development (**Figure 1C** and **Supplementary Table 2**). Among the top 50 downregulated genes, there are 49 protein-coding genes. However, the connections are not closed in the PPI network. Only 18 genes have certain correlations (**Figure 1E**).

Together, EMT, tumor angiogenesis, and collagen formation are the key characteristics of EC and CS.

### Immune Response Is More Active in CS Compared With EC

**Figure 2:**
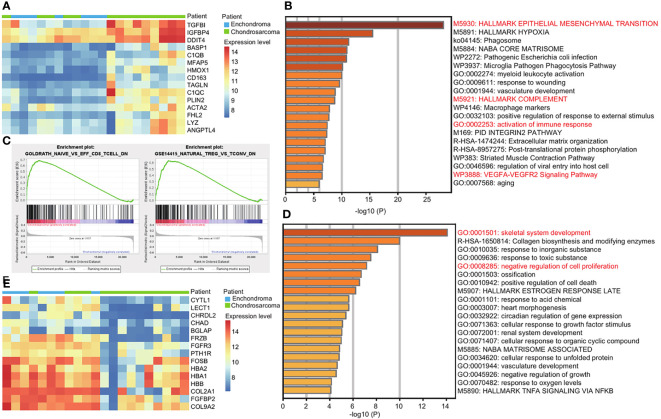
Immune response is more active in CS compared with EC. **(A)** Heatmap of the top 15 upregulated genes in CS compared with EC. Red and blue represent high and low expression levels, respectively. **(B)** Metascape enrichment analysis of the top 100 upregulated genes in CS. The color shows the p value. **(C)** GSEA map of immunologic signature gene sets in EC and CS. **(D)** Metascape enrichment analysis of the top 100 downregulated genes in CS. The color shows the p value. **(E)** Heatmap of the top 15 downregulated genes in CS compared with EC. Red and blue represent high and low expression levels, respectively.

Due to the risk of malignant transformation from EC to CS, we next explored the gene expression patterns in CS compared with EC. We used GEO2R to analyze the top 100 upregulated and downregulated genes in CS (**Supplementary Table 4**). Among the top 15 upregulated genes, TGFBI, IGFBP4, and DDIT4 are the top three upregulated genes in CS (**Figure 2A**). They promote tumor growth and metastasis (9–11). The upregulated genes are enriched in EMT and the VEGFA-VEGFR2 signaling pathway, which are important terms in **Figure 1B** (**Figure 2B**). However, the key genes are different. For example, the EMT-related genes TGFBI and BASP1 are more active in CS (**Supplementary Table 5**).

We noticed that the activation of the immune response was enriched, and the complement genes, C1QB and C1QC were two of the top 15 upregulated genes in CS (**Figures 2A, B**). We used GSEA for further investigation. In CS, a decrease in naïve CD8^+^ T cell and natural Treg cell was observed. In EC, the increase of resting T cell and naïve CD4^+^ T cell was obvious (**Figure 2C** and **Supplementary Figure 2**).

The downregulated genes are enriched in the skeletal development and negative regulation of cell proliferation (**Figure 2D**). Among the top 15 downregulated genes, CHRDL, CHAD, BGLAP, and PTH1R are related to the skeletal development. CYTL1, FRZB, and FGFR3 are related to both of the terms (**Figure 2E** and **Supplementary Table 5**).

Together, we revealed the functional differences between EC and CS. The immune response is more active in CS.

### The Unique Gene Expression Modules in CS I, and CS II

**Figure 3:**
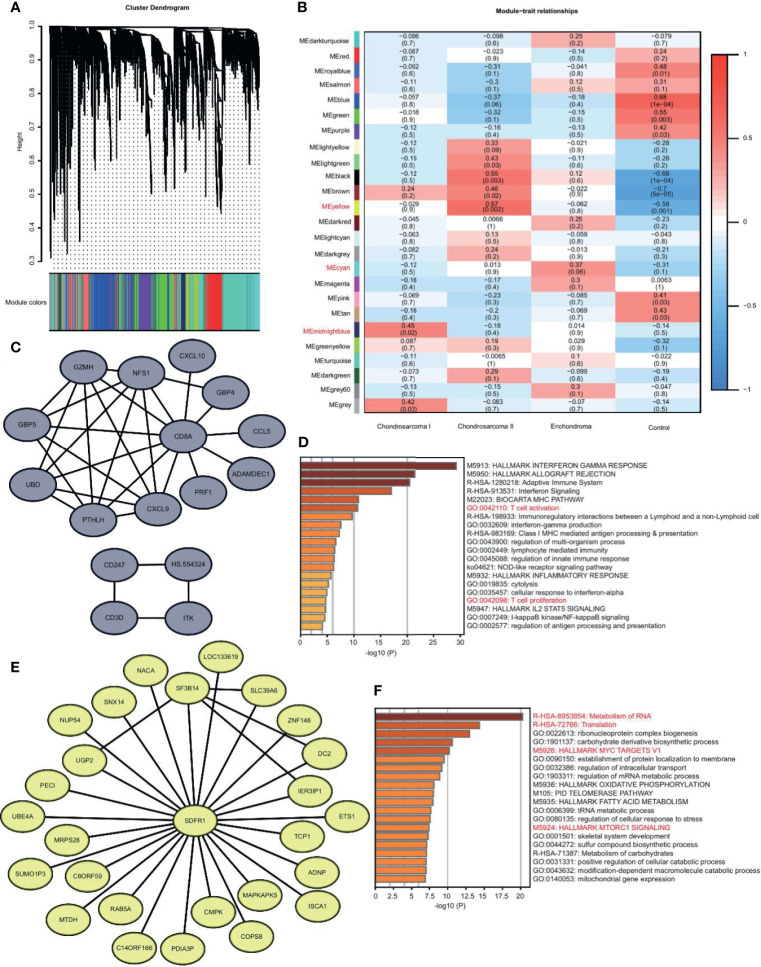
The unique gene expression modules in EC, CS I, and CS II. **(A)** Gene dendrogram obtained by average linkage hierarchical clustering. The color row underneath the dendrogram shows the module assignment. **(B)** Correlation heatmap of the modules to disease types. Each row corresponds to a module and the columns are disease types. Red and blue represent high and low correlation, respectively. The values in the cells are presented as Pearson r (p value). **(C)** Visualization of network with the genes of top-weighted connectivity in the midnight blue module. **(D)** Metascape enrichment analysis of genes in the midnight blue module. The color shows the p value. **(E)** Visualization of network with the genes of top-weighted connectivity in the yellow module. **(F)** Metascape enrichment analysis of genes in the yellow module. The color shows the p value.

CS is divided into three grades based on histopathology (1). The metastasis in Grade II and III is more frequent. In our study, we had Grade I and II patients. We used WGCNA to identify the gene modules in EC, CS I, CS II, and normal samples. We found that there were 25 modules (**Figures 3A, B**). Some modules are unique. We selected the most relevant modules and constructed the co-expression network using the genes with the top-weighted connectivity.

We were interested in the differences between CS Iand CS II. The midnight blue module is unique in CS I. The hub gene of this module is CD8A. The top-weighted genes, such as CXCL9 and CD3D, form two networks, both of which are highly correlated with T cell immunity (**Figure 3C**). The enrichment analysis of genes in this module also reveals the activation of T cells (**Figure 3D**). We believed that T cell immunity in low-grade malignancy might help avoid metastasis (12).

The yellow module is unique in CS II. The top-weighted genes form a network, and the core gene is SDFR1 (**Figure 3E**). The genes in this module are related to the translation, MYC, and MTORC1 signaling pathway (**Figure 3F**). We believed that translation disorder is more serious in high-grade malignancies, and tumor-related signaling pathways are more active (13).

Together, CS I is more likely to trigger the immunity response, and CS II succumbs to tumor regulation.

### Survival Analysis Suggesting the Prognostic Factors

**Figure 4:**
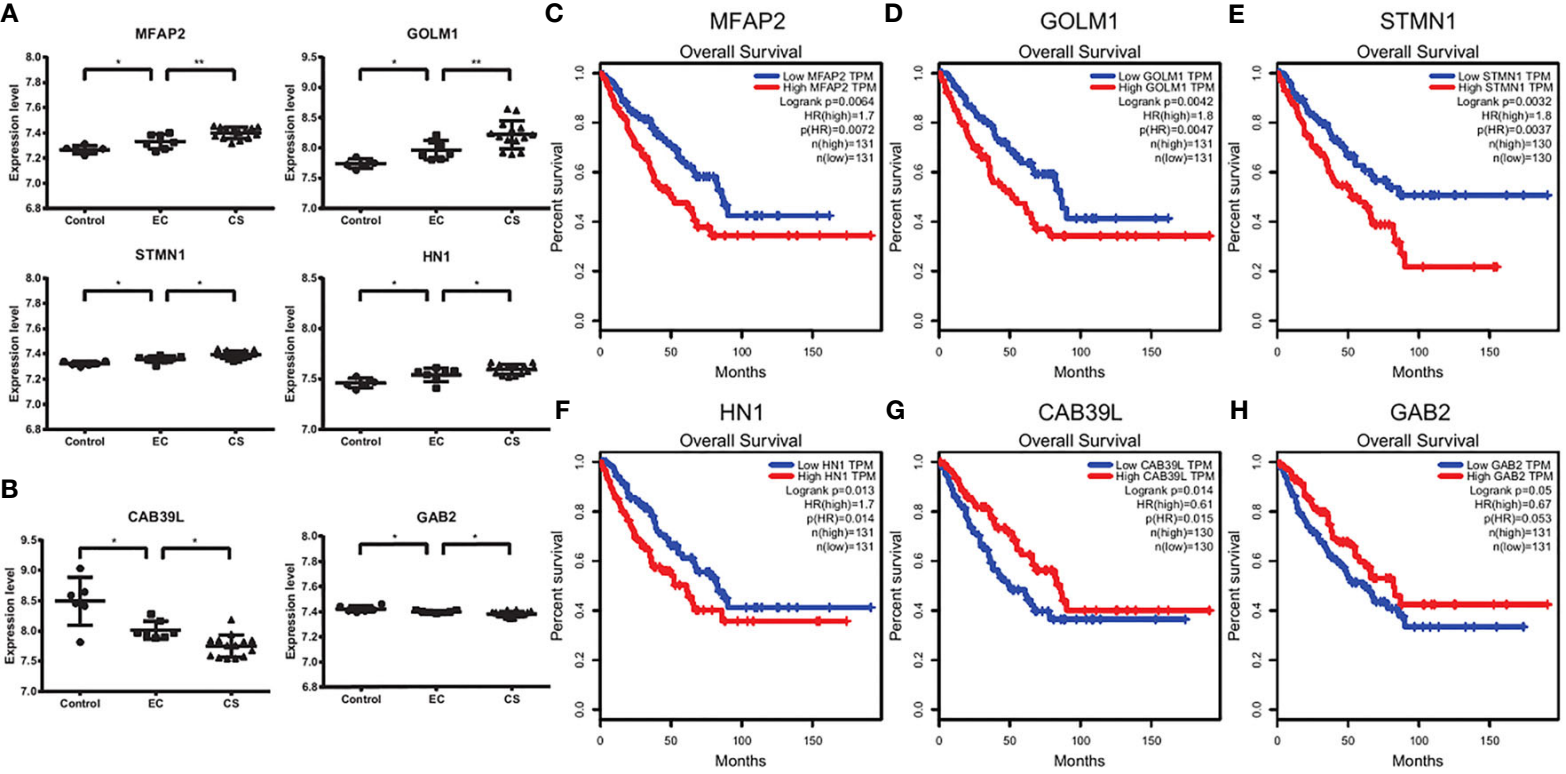
Survival analysis suggesting the prognostic factors. **(A, B)** The expression levels of MFA2, GOLM1, STMN1, HN1, CAB39L, and GAB2 in control, EC, and CS groups. Each dot represents a sample. *p < 0.05, **p < 0.01. **(C–H)** The overall survival plots using GEPIA. Red and blue represent high and low expression level groups of MFAP2 **(C)**, GOLM1 **(D)**, STMN1 **(E)**, and HN1 **(F)**, CAB39L **(G)** and GAB2 **(H)**.

We noticed that the expression levels of some genes, such as MFAP2, GOLM1(GOLPH2), STMN1, and HN1, increased continuously from control, EC, to CS, while the expression levels of some genes, such as CAB39L and GAB2 decreased (**Figures 4A, B**). We next used GEPIA and Prognoscan to explored whether these genes had potential prognostic value. There are significant differences in overall survival and distant recurrence-free survival between the high and low expression groups (**Figures 4C–H** and **Supplementary Figure 3**).

Thus, the high expression levels of MFAP2, GOLM1, STMN1, and HN1 predict a poor prognosis, while CAB39L and GAB2 predict a good prognosis.

## Discussion

EC is a benign cartilage-forming tumor with the medullary cavity of the bone (14). EC has the risk of transforming to CS. CS is a type of hyaline cartilage that forms a malignant tumor (15). In our study, we identified the DEGs in normal, EC, and CS samples. We found that EMT and the VEGFA signaling pathway were important in the initial stage. The immune response was crucial for CS, especially CS I. CS II was more active in translation, MYC, and MTOR1 signaling pathway during metastasis. Moreover, we explored some prognostic markers.

EMT may occur during different states of tumor progression (13). It plays a key role in the pathogenesis of sarcoma dedifferentiation and early dissemination (6). The EMT programs are activated by extracellular matrix components (such as collagen genes) and inflammatory stimuli (such as growth factor genes) (16). It has been reported that EMT also promotes drug resistance and may help guide precision medicine (17, 18). In our study, EC and CS had high levels of several collagen genes, and CS was associated with a higher EMT signature.

Angiogenesis is involved in tumor biology, including metastasis, metabolic deregulation, and cancer stem cell maintenance (19). The blood vessel is important for transporting nutrients and oxygen. The VEGF family is a key mediator of angiogenesis (7). Blocking VEGFA is used to treat tumors (20). In one study, patients benefited from Ramncirumab, a monoclonal antibody that binds to VEGFR-2. It blocks the downstream effects of the VEGF pathway in angiogenesis (21). In another research, Bevacizumab, a monoclonal antibody targeting VEGF receptor, has been tested in a phase II clinical trial (22). In our study, the VEGFA-VEGFR2 signaling pathway was active in EC and CS. It gives us a hint that the anti-angiogenesis may be a powerful treatment to prevent recurrence.

Immunomodulatory drugs and immunotherapeutic agents have been used to treat various tumors (23). These approaches target the specific antigens expressed on tumor cells and induce cell death through tumor-infiltrating T cells (24). However, the heterogeneity in CD8^+^ T cell infiltration governs differential immunity (25). It usually acts as a cytotoxic cell that kills tumor cells but loses its effector function. Other cytokines and chemokines are important in regulating the antitumor response of CD8^+^ T cells (26). Treg cell reduces the expansion of CD8^+^ T cell (27). CXCL9 contributes to the recruitment and infiltration of CD8^+^ T cell (28). In our study, the upregulation of effect CD8^+^ T cells and the downregulation of Treg cells were observed in CS. Interestingly, CS I, not CS II, had the CD8 module, which was associated with CXCL9 in the network. In addition, it indicated the depletion of T cells in the late state.

In our study, some genes increased or decreased continuously from control, EC to CS. MFAP2, an extracellular matrix glycoprotein, is specifically expressed in osteoblastic-like cells (29). GOLM1 participates in immune regulation and the promotes EMT. It is a promising marker for early diagnosis and prognosis of hepatocellular carcinoma (30). In a study of 4625 patients with solid tumors, overexpression of STMN1 was associated with poor overall survival (31). HN1, interacting with STMN1, reduces α-tubulin acetylation and promotes tumor progression through EMT (32). CAB39L is a tumor metabolism regulator with the functions of tumor suppressor (33). It has been reported that GAB2 promotes tumor cell metastasis, migration, and recurrence (34). However, in our study, the expression level of GAB2 was reduced in CS. With the exception of STMN1, all of the above genes were first discussed as prognostic factors in sarcoma. Although the underlying mechanisms in EC and CS are not yet fully understood, we believe that MFAP2, GOLM1, STMN1, and HN1 are markers for poor prognosis, while CAB39L and GAB2 indicate a good prognosis.

In conclusion, we identified the DEGs in EC, CS and normal controls. We found that EMT and angiogenesis were active in CS. The CD8^+^ T cell immunity was enhanced in CS I. We suggested some prognostic factors. Our study provided new ideas for the diagnosis and treatment of EC and CS.

## Data Availability Statement

The original contributions presented in the study are included in the article/**Supplementary Material**. Further inquiries can be directed to the corresponding authors.

## Author Contributions

HH, GG, and XH designed the study. JW, YH, CY, XL, LW, JH, and DL performed analysis and interpretation. JW and TH wrote the manuscript. All authors contributed to the article and approved the submitted version.

## Funding

This work was supported by grants from the Key R&D Project of Zhejiang Science and Technology Department (grant No. 2020C03G2013586), the Natural Science Foundation of China (grant No. 81730008), Key Project of Science and Technology Department of Zhejiang Province (grant No. 2019C03016).

## Conflict of Interest

The authors declare that the research was conducted in the absence of any commercial or financial relationships that could be construed as a potential conflict of interest.

## Publisher’s Note

All claims expressed in this article are solely those of the authors and do not necessarily represent those of their affiliated organizations, or those of the publisher, the editors and the reviewers. Any product that may be evaluated in this article, or claim that may be made by its manufacturer, is not guaranteed or endorsed by the publisher.

## Supplementary Material

Supplementary Figure 1 DEGs in other sarcoma datasets confirm the activation of EMT and angiogenesis. **(A, B)** Metascape enrichment analysis for viewing the enrichment terms of upregulated genes in Ewing sarcoma **(A)** and leiomyosarcoma **(B)** patients. The genes with p < 0.01 and logFC>1 are considered as the upregulated genes. The top 100 upregulated genes were used for the enrichment analysis. The color shows the p value.

Supplementary Figure 2 GSEA map of EC and CS. GSEA of immunologic signature gene sets in EC and CS.

Supplementary Figure 3 The distant recurrence-free survival suggesting the prognostic factors. **(A-F)** The distant recurrence-free survival using Prognoscan. Red and blue represent high and low expression level groups of MFAP2 **(A)**, GOLM1 **(B)**, STMN1 **(C)**, and HN1 **(D)**, CAB39L **(E)** and GAB2 **(F)**.
