# Supplementary material for: The Key Gene Expression Patterns and Prognostic Factors in Malignant Transformation from Enchondroma to Chondrosarcoma
Source: Front Oncol. 2021 Sep 10;11:693034. doi: 10.3389/fonc.2021.693034 (PMC8461174; doi:10.3389/fonc.2021.693034)
Supplement: Supplementary Figure 1 — DEGs in other sarcoma datasets confirm the activation of EMT and angiogenesis. (A, B) Metascape enrichment analysis for viewing the enrichment terms of upregulated genes in Ewing sarcoma (A) and leiomyosarcoma (B) patients. The genes with p < 0.01 and logFC>1 are considered as the upregulated genes. The top 100 upregulated genes were used for the enrichment analysis. The color shows the p value. [file DataSheet_1.pdf]

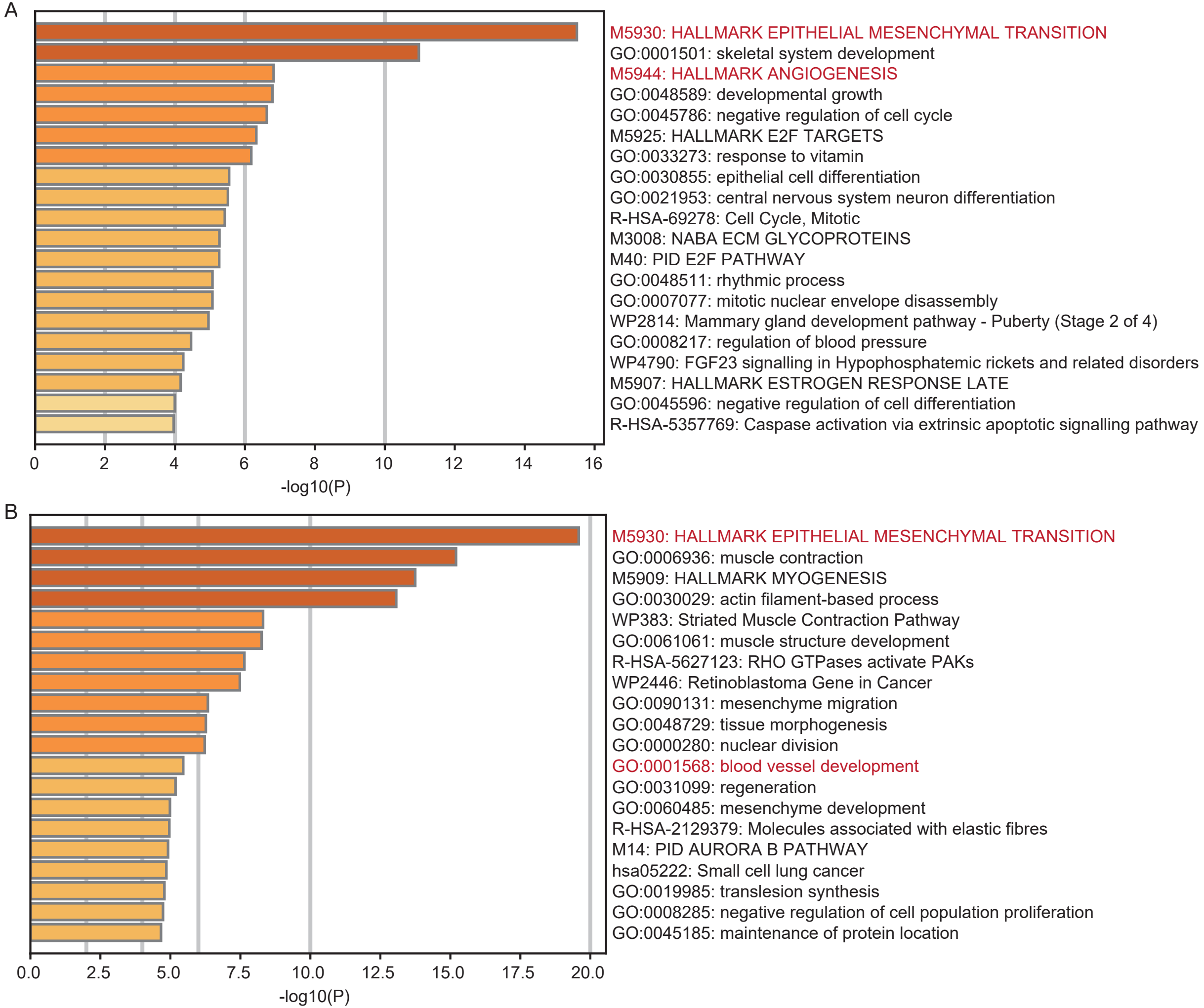

A
M5930: HALLMARK EPITHELIAL MESENCHYMAL TRANSITION
GO:0001501: skeletal system development
M5944: HALLMARK ANGIOGENESIS
GO:0048589: developmental growth
GO:0045786: negative regulation of cell cycle
M5925: HALLMARK E2F TARGETS
GO:0033273: response to vitamin
GO:0030855: epithelial cell differentiation
GO:0021953: central nervous system neuron differentiation
R-HSA-69278: Cell Cycle, Mitotic
M3008: NABA ECM GLYCOPROTEINS
M40: PID E2F PATHWAY
GO:0048511: rhythmic process
GO:0007077: mitotic nuclear envelope disassembly
WP2814: Mammary gland development pathway - Puberty (Stage 2 of 4)
GO:0008217: regulation of blood pressure
WP4790: FGF23 signalling in Hypophosphatemic rickets and related disorders
M5907: HALLMARK ESTROGEN RESPONSE LATE
GO:0045596: negative regulation of cell differentiation
R-HSA-5357769: Caspase activation via extrinsic apoptotic signalling pathway
0 2 4 6 8 10 12 14 16
-log10(P)
B
M5930: HALLMARK EPITHELIAL MESENCHYMAL TRANSITION
GO:0006936: muscle contraction
M5909: HALLMARK MYOGENESIS
GO:0030029: actin filament-based process
WP383: Striated Muscle Contraction Pathway
GO:0061061: muscle structure development
R-HSA-5627123: RHO GTPases activate PAKs
WP2446: Retinoblastoma Gene in Cancer
GO:0090131: mesenchyme migration
GO:0048729: tissue morphogenesis
GO:0000280: nuclear division
GO:0001568: blood vessel development
GO:0031099: regeneration
GO:0060485: mesenchyme development
R-HSA-2129379: Molecules associated with elastic fibres
M14: PID AURORA B PATHWAY
hsa05222: Small cell lung cancer
GO:0019985: translesion synthesis
GO:0008285: negative regulation of cell population proliferation
GO:0045185: maintenance of protein location
0.0 2.5 5.0 7.5 10.0 12.5 15.0 17.5 20.0
-log10(P)
