# Supplementary material for: The Key Gene Expression Patterns and Prognostic Factors in Malignant Transformation from Enchondroma to Chondrosarcoma
Source: Front Oncol. 2021 Sep 10;11:693034. doi: 10.3389/fonc.2021.693034 (PMC8461174; doi:10.3389/fonc.2021.693034)
Supplement: Supplementary Figure 2 — GSEA map of EC and CS. GSEA of immunologic signature gene sets in EC and CS. [file DataSheet_2.pdf]

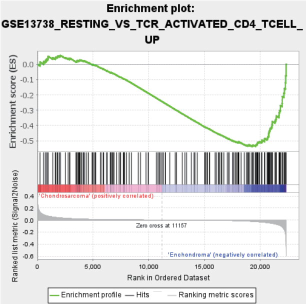
Enrichment plot:
GSE13738_RESTING_VS_TCR_ACTIVATED_CD4_TCELL_UP
Enrichment score (ES)
0.0
-0.1
-0.2
-0.3
-0.4
-0.5
'Chondrosarcoma' (positively correlated)
Zero cross at 11157
'Enchondroma' (negatively correlated)
Ranked list metric (Signal2Noise)
0.4
0.2
0.0
-0.2
-0.4
-0.6
0
5,000
10,000
15,000
20,000
Rank in Ordered Dataset
Enrichment profile
Hits
Ranking metric scores

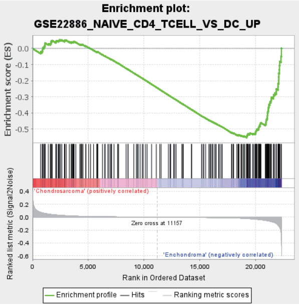
Enrichment plot:
GSE22886_NAIVE_CD4_TCELL_VS_DC_UP
Enrichment score (ES)
0.0
-0.1
-0.2
-0.3
-0.4
-0.5
'Chondrosarcoma' (positively correlated)
Zero cross at 11157
'Enchondroma' (negatively correlated)
Ranked list metric (Signal2Noise)
0.4
0.2
0.0
-0.2
-0.4
-0.6
0
5,000
10,000
15,000
20,000
Rank in Ordered Dataset
Enrichment profile
Hits
Ranking metric scores
