# Supplementary figures and images for: The Key Gene Expression Patterns and Prognostic Factors in Malignant Transformation from Enchondroma to Chondrosarcoma
Source: Front Oncol. 2021 Sep 10;11:693034. doi: 10.3389/fonc.2021.693034 (PMC8461174; doi:10.3389/fonc.2021.693034)

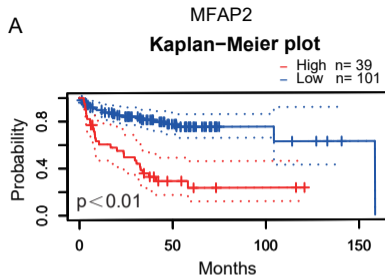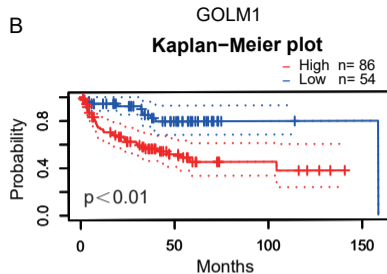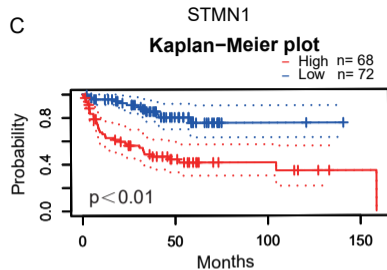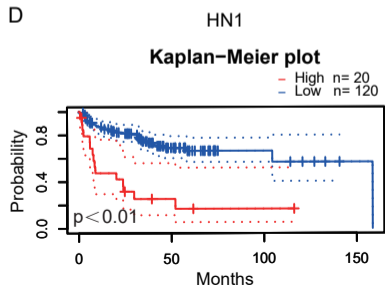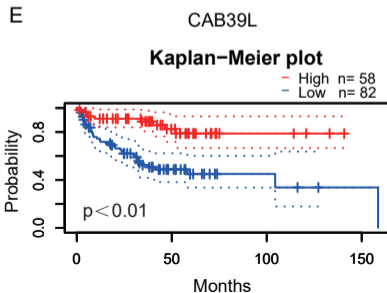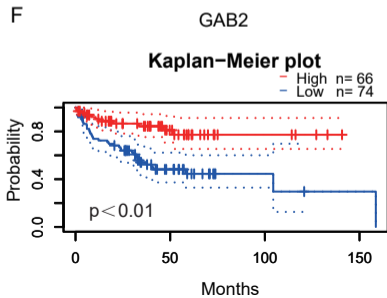

Supplement: Supplementary Figure 3 — The distant recurrence-free survival suggesting the prognostic factors. (A-F) The distant recurrence-free survival using Prognoscan. Red and blue represent high and low expression level groups of MFAP2 (A), GOLM1 (B), STMN1 (C), and HN1 (D), CAB39L (E) and GAB2 (F). [file DataSheet_3.pdf]
